# Non-Small-Cell Lung Cancer: New Rare Targets—New Targeted Therapies—State of The Art and Future Directions

**DOI:** 10.3390/cancers13081829

**Published:** 2021-04-12

**Authors:** Katarzyna Stencel, Izabela Chmielewska, Janusz Milanowski, Rodryg Ramlau

**Affiliations:** 1 Oncology Clinic, Poznan University of Medical Sciences, 61-701 Poznań, Poland; rodryg.ramlau@skpp.edu.pl; 2 Department of Chemotherapy, Clinical Hospital of Lord’s Transfiguration, 60-569 Poznan, Poland; 3 Chair and Department of Pneumonology, Oncology and Allergology, Medical University of Lublin, 20-059 Lublin, Poland; izachm@wp.pl (I.C.); janusz.milanowski@umlub.pl (J.M.)

**Keywords:** lung cancer, gene alterations, targeted therapy, *MET* amplification, *MET* skipping mutation, *NTRK* fusions, *RET* mutations, *HER2* mutations, exon 20 insertions

## Abstract

**Simple Summary:**

The use of novel therapeutic drugs in lung cancer has changed the paradigm of the diagnosis and treatment of lung cancer. Due to the development of advanced diagnostic procedures (e.g., next generation sequencing (NGS)) around half of non-small-cell lung cancer (NSCLC) patients can be identified with genetic aberrations. The presence of activating mutations of *EGFR*, *ALK* and *ROS-1* have already been well explored. New targets that can be successfully targeted include *NTRK*, *MET*, *RET* and *HER 2* genes. Some particles have already received FDA approval, whereas many more are in the late stages of clinical trials. Considering rapid changes in thoracic oncology, an up-to-date summary is needed. In this review, we present the current landscape of approved therapeutic drugs, as well as important ongoing clinical trials.

**Abstract:**

Lung cancer is the most common cause of cancer-related death worldwide, and the prognosis for stage IV remains poor. The presence of genetic alterations in tumor cells, such as *EGFR* and *BRAF* gene mutations, as well as *ALK* and *ROS1* gene rearrangements, are indications for targeted therapies. Many such treatments are already registered and used on a wide scale. In comparison to standard chemotherapy, they can prolong not only progression-free survival but also overall survival. Moreover, they are able to provide excellent quality of life and rapid improvement of cancer-related symptoms such as dyspnea, cough and pain. Recent years have witnessed great advances in both molecular diagnostics and new molecular therapies for non-small-cell lung cancer. This review presents new therapeutic targets in NSCLC, as well as drugs of which the activity against *NTRK*, *RET*, *MET* or *HER2* gene alterations (including *EGFR* exon 20 insertions) has either been confirmed or is currently being evaluated. Although these particular genetic alterations in NSCLC are generally rare, each accounting for 1–2% of patients, in total about half of all patients have molecular alterations and may ultimately receive targeted therapies.

## 1. Introduction

Lung cancer is the most common cause of cancer-related death worldwide, and the prognosis in stage IV remains poor. Approximately 85% of cases are patients with non-small-cell lung cancer (NSCLC); according to the estimates of the World Health Organization (WHO), 1.8 million patients per year receive this diagnosis [1]. The most frequent subtype of NSCLC is adenocarcinoma, most commonly peripheral, which more often affects non-smokers, light smokers or former smokers. Younger individuals, below the age of 45, are diagnosed almost exclusively with adenocarcinoma; in such patients, this diagnosis is more often associated with the presence of molecular alterations (driver mutations). The primary treatment for stage IV patients who are not eligible for targeted therapies or immunotherapy continues to consist in platinum-based chemotherapy (cisplatin/carboplatin); the length of their survival usually does not exceed 12 months [2]. The introduction of immunocompetent agents as the second line of treatment with nivolumab or atezolizumab and, subsequently, as the first line of treatment in combination with chemotherapy, has improved the prognosis for NSCLC patients. Applying nivolumab or atezolizumab in second line treatment regardless PD-L1 status instead of docetaxel may bring an advantage of about 3 months in overall survival (OS). Similarly, the use of pembrolizumab instead of platinum-based chemotherapy in first line treatment prolongs survival in PD-L1 high NSCLC (PD-L1 > 50%). However, the most spectacular therapeutic effects are achievable only with the use of molecularly targeted agents in patients with particular molecular alterations. In comparison to standard chemotherapy, these agents can not only increase overall survival (OS), but can also result in significantly longer progression-free survival (PFS), typically lengthening the time during which the patient remains free from the disease’s symptoms and the subsequent deterioration, as well as improving the patient’s quality of life (QoL) by improving their clinical condition relatively quickly. The most common molecular alterations, found in approximately 10–15% of Caucasian patients, are mutations in the epidermal growth factor receptor gene (*EGFR*). They occur more often in patients suffering from lung adenocarcinoma, women, Asian patients (40–45% of patients) and in non-smokers/light smokers [3,4]. In patients with an activating mutation in the *EGFR* gene, the first line of treatment may include first-generation inhibitors (erlotinib, gefitinib), second-generation inhibitors (afatinib, dacomitinib), or the third-generation inhibitor osimertinib, which can also be employed if the patient develops a resistance mutation (T790M) while being treated with first- or second-generation inhibitors [5,6,7,8,9,10]. Osimertinib was also approved by the U.S. Food and Drug Administration (FDA) as a 3-year adjuvant therapy in patients with common activating mutations in the *EGFR* gene (exon 19 deletions or L858R substitutions) after radical thoracic surgery procedures [11]. Osimertinib has good blood–brain barrier penetration, which reduces the risk of intracranial dissemination [12]. Another molecular alteration commonly treated with targeted therapies is rearrangement in the anaplastic lymphoma kinase gene (*ALK*), which is diagnosed in approximately 5–7% of NSCLC patients, mostly young individuals with the signet-ring-cell subtype of lung adenocarcinoma [13]. Importantly, patients with molecular alterations are especially likely to have metastases to the central nervous system (CNS)—CNS metastases are found already at diagnosis in approximately 40% of patients [14]. The current recommendation is to begin treatment not with first-generation inhibitors (crizotinib) but with second-generation inhibitors (alectinib, brigatinib, ceritinib), which have been demonstrated to result not only in longer PFS but also in lower rates of CNS metastasis development and progression [15,16]. ALK tyrosine kinase inhibitors (TKIs) can be used sequentially, and patients in whom progression is observed during treatment with a second-generation inhibitor can be treated with a third-generation inhibitor—lorlatinib. Patients with ROS1-positive NSCLC (1–2% of NSCLC patients) and BRAF-positive NSCLC (2–4% of NSCLC patients) are also commonly treated with targeted therapies: crizotinib for ROS1-rearranged NSCLC and dabrafenib with trametinib for BRAF-mutant NSCLC [17,18]. The introduction of a targeted therapy is conditional on the acquirement of a sufficiently large tissue sample of appropriate quality and requires molecular analysis to be conducted. The optimal method for the latter is next-generation sequencing (NGS), which saves both time and the precious tissue material (assessing all genes at once, not one by one). Importantly, introducing NGS into clinical practice creates an opportunity to detect rare alterations such as the *MET* exon14 skipping mutation (3–4% of NSCLC patients), *RET* gene fusions (1–2% of NSCLC patients), *NTRK* gene fusions (approximately 1% of NSCLC patients) and exon 20 insertions of *EGFR* gene (1–2%) or *HER2* gene mutations (2–4% of NSCLC patients). Although these particular genetic alterations in NSCLC are generally rare, about half of the all NSCLC patients have molecular alterations and may ultimately receive targeted therapies.

**Figure 1:**
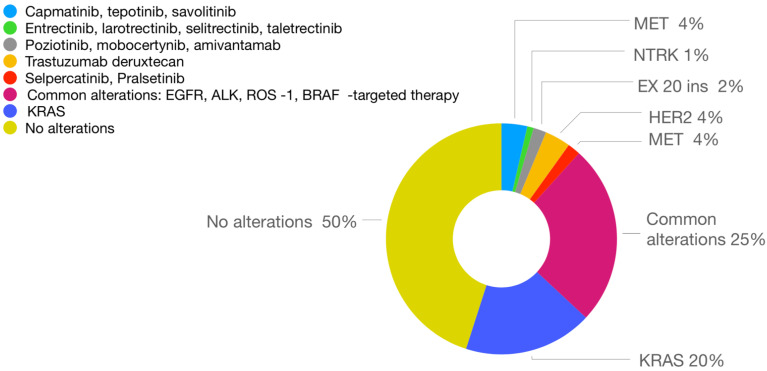
Frequency of genomic alterations in non-small-cell lung cancer (NSCLC) and corresponding targeted therapies.

This study reviews the available therapeutic options and the molecules that appear promising based on the results of clinical studies and which may soon be available for patients with rare molecular alterations such as mutations or rearrangements in the *MET*, *RET* and *NTRK* genes, as well as for patients with insertions in exon 20 of the *EGFR* and *HER2* genes. The frequency of these alterations and the available targeted therapies are shown in Figure 1.

## 2. Results

### 2.1. Met Pathway Inhibitors

The *MET* gene is a proto-oncogene which encodes the c-Met protein (a receptor tyrosine kinase) of which the ligand is the hepatocyte growth factor (HGF). The binding of the ligand to the receptor initiates a signaling cascade of the RAS-RAF, STAT3 and PI3K pathways. Abnormal activation of the mesenchymal epithelial transition (MET) pathway can be caused by overexpression or amplification of the gene or by a skipping mutation in exon 14 of the *MET* gene [19,20]. In molecular biology, exon skipping is a form of RNA splicing used to cause cells to “skip” over misaligned or faulty exons, leading to a truncated but still functional protein despite the genetic mutation. A *MET* exon 14 skipping mutation results in MET ubiquitination, reduced MET turnover and the activation of cellular signal transmission. Abnormal activation of the MET pathway increases the proliferation of neoplastic cells, their longevity, invasiveness and ability to metastasize [21,22]. Skipping mutations in exon 14 of the *MET* gene are rare, occurring in 3–4% of NSCLC patients, and their presence usually precludes the presence of other driver mutations [23,24]. They are most often encountered in smokers and patients diagnosed with lung adenocarcinoma. They should also be routinely sought in patients with pulmonary sarcomatoid carcinoma (PSC), as their prevalence in this patient group exceeds 7% [25]. In contrast to most driver mutations occurring in lung cancer, they are most commonly observed in elderly patients (after the age of 70) [26]. It is associated with unfavorable prognosis [27,28] and inefficacy of standard NSCLC treatments, including immunotherapy [29,30]. The percentage of patients with CNS metastases at diagnosis amounts to approximately 17%. Over the course of the disease, metastases may appear in as many as 36% of patients. Neoplastic involvement of the meninges [31] is observed in approximately 17% of these patients. MET amplification occurs in 1–6% of patients with NSCLC [32,33].

Non-selective MET TKIs include, among others, crizotinib and foretinib, whereas selective MET TKIs include tivantinib, savolitinib, capmatinib and tepotinib.

#### 2.1.1. Capmatinib

The efficacy and safety of capmatinib (INC280) in patients with NSCLC with the presence of a skipping mutation in exon 14 of the *MET* gene or a *MET* gene amplification was assessed in the open-label phase 2 clinical study GEOMETRY mono-1 [34]. The study included 364 patients (including 97 patients in whom a skipping mutation was found in the *MET* gene). According to the study protocol, patients with brain metastases were eligible to participate. The study’s primary endpoint was the overall response rate (ORR), i.e., the percentage of patients in whom complete or partial response to treatment was observed. Secondary endpoints included the duration of response (DOR), the disease control rate (DCR; the percentage of patients showing not only complete or partial response to the treatment but also stabilization of the disease) and treatment safety. Patients with skipping mutations and patients with amplifications of the *MET* gene were assigned to cohorts in which no systemic treatment had been previously employed or to cohorts in which patients had received one or two lines of prior systemic treatment. The treatment results were better in the group of patients who received capmatinib as the first line of therapy, which suggest that targeted therapies should be applied immediately after diagnosis, in first line treatment. In most cases, a response to treatment was observed as early as during the first evaluation of the treatment’s efficacy. In patients with *MET* exon 14 skipping mutations, the ORR amounted to 41% in the group who had received prior systemic therapy and 68% among patients who had not been previously treated. Importantly, capmatinib is characterized by high intracranial activity. Among the patients in whom measurable CNS lesions were found, disease control was observed in 12 out of 13 patients, and response to treatment was observed in seven out of 13 patients. In patients with *MET* amplification, capmatinib’s efficacy was only confirmed in patients with a gene copy number of at least 10. The ORR amounted to 29% of previously treated patients and 40% for patients who had not received prior systemic treatment. The detailed results of capmatinib’s efficacy are presented in the Table 1. The most frequent adverse effects of capmatinib included peripheral edemas, nausea, vomiting and reversible elevations in creatinine concentration associated with the inhibition of renal transporter activity and glomerular filtration. Severe adverse events related to the treatment occurred in 13% of the patients treated with capmatinib.

#### 2.1.2. Tepotinib

Tepotinib is another selective MET TKI with confirmed efficacy in patients with a *MET* exon 14 skipping mutation. Its efficacy and safety were assessed in the phase 2 clinical trial VISION [35]. The study included 152 patients (cohort A: patients with exon 14 mutations; cohort B: patients with *MET* gene amplification). To date, only the results of cohort A have been made available after a period of follow-up lasting at least 9 months. The study included patients in whom skipping mutations were found based on circulating free DNA (cfDNA) obtained from plasma (liquid biopsy, n = 66) or tumor samples (tissue biopsy, n = 60). The study’s protocol permitted the presence of neurologically stable metastases to the CNS and no more than two lines of previous systemic therapy. As in the case of GEOMETRY mono-1, the study’s primary endpoint was ORR as assessed by an independent committee. The molecular response to treatment was also assessed. A complete response was defined as the disappearance of all cfDNA with MET exon 14 mutations; a deep molecular response was defined as a depletion of its amount by more than 75% but less than 100%. The ORR was similar regardless of the type of samples in which the mutation was revealed (46% in the whole group, 48% in liquid samples and 50% in tissue samples). This is very valuable information, because it means that liquid biopsy could be successfully used when a tissue sample is not available. The results were also similar regardless of the line of treatment in which tepotinib was used, and a response to treatment was usually noted as early as after 6 weeks from the commencement of treatment. The median DOR was 11.1 months, 9.9 months and 15.7 months for the whole group, the liquid biopsy group and the tissue biopsy group, respectively. The presence of CNS metastases was noted in 55% of the patients. A molecular response to treatment was confirmed in 67% of the patients; a radiological response to treatment was also confirmed in 71% of these patients. The most frequent adverse effects of tepotinib were peripheral edemas (63% of the patients; grade 3 or higher according to CTCAE (Common Terminology Criteria for Adverse Events) in 7% of the patients), nausea (26%), diarrhea (22%) and elevated concentrations of creatinine (18%). Other, less frequent, adverse effects included pleural effusion (8%) and increased activity of amylase (11%) and lipase (9%). The detailed results of tepotinib’s efficacy are presented in Table 1.

#### 2.1.3. Savolitinib

Savolitinib (AZD6094, HMPL-504, volitinib) is a highly selective MET TKI. Its efficacy in NSCLC patients was assessed in a multicenter phase 2 clinical trial [36]. The study included 87 patients with the presence of a skipping mutation in the *MET* gene; 70 of these patients received treatment. Approximately 57% of the patients were diagnosed with lung adenocarcinoma, and nearly 37% had PSC. Among the patients treated with savolitinib, the ORR amounted to 47.5% and the DCR was as high as 93.4%. The median PFS in the whole group was 6.8 months, and the median DOR was yet to be determined at the time of publication. The prognosis for the PSC patients was significantly worse—the median PFS was 5.5 months as compared to 9.7 months for the other subtypes of NSCLC. The most frequent adverse events were similar to those observed with other MET TKIs; they included peripheral edemas, nausea and vomiting, as well as increased aminotransferase activity and hypoalbuminemia. The detailed results of savolitinib’s efficacy are presented in Table 1.

### 2.2. Ntrk Pathway Inhibitors

The detection of *NTRK* (neurotrophic tyrosine kinase receptor) gene fusions grew in significance with the availability of TKIs. Owing to the use of NGS, *NTRK* gene alterations have been identified in 19 different types of neoplasms [37]. *NTRK* gene fusions involving *NTRK1*, *NTRK2* and *NTRK3* (encoding receptor proteins TRKA, TRKB and TRKC, respectively) are responsible for many neoplasms in both adults and children, including rare cancers such as secretory breast carcinoma and infantile fibrosarcoma [38]. The prevalence of *NTRK* gene fusions among patients with non-squamous NSCLC patients is approx. 1%; this figure rises to approx. 3% in the population of patients diagnosed with adenocarcinoma. The presence of molecular aberrations such as *EGFR* gene mutations or *ALK* or *ROS1* rearrangements is typically mutually exclusive with *NTRK* fusions, which makes it easier to narrow down diagnostic investigations and broadens the available therapeutic options [39]. Therefore, one of the preferred diagnostic schemes is genomic-based triage. In other words, if single diagnostic tests are being used, they should cover the most frequent aberration first. In the context of NSCLC, this means that *NTRK* fusions are sought after the exclusion of other, more frequent, driver mutations [40]. As of yet, the clinical characteristics of patients with *NTRK* fusions have not yet been determined. Nevertheless, it is important to note that treating *NTRK* fusion-positive patients with a NTRK inhibitor can yield high rates of response regardless of the tumor’s histology, the patient’s age or the type of fusion. For this reason, larotrectinib and entrectinib are considered to be tissue-agnostic and have been approved by the FDA for the treatment of patients with *NTRK* fusions regardless of the histopathological type involved. Clinical studies indicate that molecularly selected NSCLC patients with *NTRK* fusions also obtain significant clinical benefits from therapies targeting TRK. Unfortunately, most tumors ultimately develop resistance to first-generation NTRK inhibitors such as larotrectinib, despite showing an initial response to treatment. This has prompted an intensive search for new molecules that could potentially exhibit activity in the presence of acquired resistance. Repotrectinib and selitrectinib have shown efficacy in preclinical studies and in small patient samples in early phase trials in cases involving progression depending on the TRK pathway [41]. The detailed results of NTRK TKI efficacy are presented in the Table 2.

#### 2.2.1. Entrectinib

Entrectinib is the best studied oral inhibitor of TRKA, TRKB, TRKC, ROS1 and ALK. Entrectinib exerts its antineoplastic action by inhibiting the phosphorylation of TRK fusion proteins and signaling molecules for TRK. The benefits of entrectinib were evaluated in single-arm studies assessing a relatively small sample of patients with tumors harboring *NTRK* gene fusions. The positive effects of the drug were demonstrated based on the overall response rate and the duration of the response. The efficacy and safety of entrectinib were assessed based on the analysis of three phase 1 or 2 clinical trials (ALKA-372-001, STARTRK-1, STARTRK-2) [42]. The studies included 54 patients with *NTRK* fusion-positive solid tumors. In this group, 19% of the patients had NSCLC and more than half had CNS metastases. Among the NSCLC patients (n = 10), the overall response rate amounted to 70% (complete response: 10%, partial response: 60%), and the median PFS was 14.9 months (95% CI, 4.7 months to not estimable). Most of the adverse events noted among the patients treated with entrectinib were grade 1 or 2 events. The most common ones included: dysgeusia (47% of patients), constipation (28%), fatigue (28%), diarrhea, peripheral edemas, dizziness, paresthesias and nausea/vomiting. Based on these results, entrectinib was approved for the treatment of patients with *NTRK* fusion-positive solid tumors by the Japanese Ministry of Health, Labor and Welfare and by the FDA (in June and August of 2019, respectively).

#### 2.2.2. Larotrectinib

The activity of larotrectinib in the treatment of *NTRK* fusion-positive solid tumors was demonstrated in three phase 1 and/or 2 clinical studies (NCT02122913, NCT02637687 and NCT02576431). The studies evaluated 153 patients with solid tumors, including NSCLC tumors [43]. An objective response was achieved in 121 patients according to investigator assessment (79%, 95% CI 72–85); complete response was achieved in 24 cases (16%). Median DOR was 35.2 months, and the median PFS was 28.3 months. Responses were achieved regardless of the histopathological type of the tumor, the type of the gene fusion or patient age. Among the 12 NSCLC patients, an objective response was achieved in nine patients (75%, 43–95). In the expanded safety population, 260 patients treated regardless of their *TRK* fusion status were analyzed; the most frequent grade 3 and 4 adverse events related to larotrectinib were increased alanine aminotransferase activity (eight (3%) out of 260 patients), anemia (six (2%)) and reduced neutrophil count (five (2%)). Treatment was discontinued due to adverse events related to the agent only in 2% of the whole population [43]. Based on these results, larotrectinib was approved by the FDA for the treatment of adult and pediatric patients with solid tumors exhibiting *NTRK* gene fusions.

#### 2.2.3. Selitrectinib (LOXO-195)

Selitrectinib (LOXO-195) is another next-generation TRK inhibitor. Its chemical structure is similar to that of larotrectinib. Selitrectinib’s potent activity against acquired resistance mutations in the TRK kinase domain was demonstrated in enzyme and cell-based assays as well as in in vivo tumor models [44]. In a phase 1 clinical trial in which 31 patients received selitrectinib, the ORR amounted to 34% [45]. The response rate was higher (45%) in the group of patients progressing in the TRK mechanism (with a confirmed secondary resistance mutation). The high response rates noted in patients previously treated with NTRK inhibitors offer the chance of prolonging survival with the use of sequential therapies.

#### 2.2.4. Repotrectinib

Repotrectinib is a next-generation inhibitor tested for molecular alterations in the genes *ALK*, *ROS1* and *NTRK*; its activity was evaluated in the phase 1 clinical trial TRIDENT-1 [46,47]. The study analyzed patients with solid tumors, with NSCLC patients comprising approx. 83% of the group. The study included eight patients with *NTRK* gene fusions. The treatment’s good activity and tolerance was also demonstrated in the group of patients with *NTRK* gene fusions who had been previously treated with first-generation inhibitors (entrectinib or larotrectinib). Three of these patients responded to treatment, resulting in an ORR of 50%. The initial safety data from 83 patients treated with various doses of repotrectinib (from 40 mg per day to 200 mg twice per day), indicating that the drug’s toxicity profile is relatively safe. The most frequent adverse events related to the treatment include dizziness (57%), dysgeusia (51%), dyspnea (30%) and fatigue (30%). Repotrectinib restores the ability to control the disease when progression occurs through the mediation of the kinase domain, despite the acquired resistance to first-generation TRK inhibitors.

#### 2.2.5. Taletrectinib (DS-6051b/AB-106)

Taletrectinib (DS-6051b/AB-106) was designed as a new highly selective inhibitor of the ROS1 and NTRK kinases. In June 2020, the results of a phase 1 trial (NCT02279433) were published [48]. The study included adult patients with solid tumors with documented *ROS1* or *NTRK* rearrangement. Teletrectinib exhibited manageable toxicity. The maximum tolerated dose was established at 800 mg per day. The ORR was 33.3% among the six patients with crizotinib-refractory ROS1-positive NSCLC. However, there are no data on the drug’s efficacy in NTRK-positive NSCLC. Further studies are planned to assess the drug’s efficacy in solid tumors with *NTRK* gene fusions.

### 2.3. Agents Active in Patients with Insertions in Exon 20 of the Egfr or Her2 Gene

Insertions in exon 20 of the *EGFR* gene constitute 4–10% of all *EGFR* gene mutations [49,50] and occur in 1–2% of NSCLC patients. Like other activating mutations in the *EGFR* gene, their frequency is higher among women, Asian patients, patients diagnosed with lung adenocarcinoma and non-smoking patients; they are also mutually exclusive with other molecular alterations [50,51]. Like other activating mutations, exon 20 insertions also cause constitutive activation of tyrosine kinase, but most registered EGFR TKIs are not effective in the discussed group of patients. *EGFR* exon 20 insertions form a heterogeneous group of molecular alterations, which includes insertions or duplications between the 3rd and 21st base pair involving from one to seven amino acids located between amino acids in positions 762 and 774 of the EGFR protein (D761-C775) [50]. The three most common subtypes of *EGFR* exon 20 insertions are D770-N771 insX (25.5% of all exon 20 insertions), V769-D770 insX (24.6%) and H773-V774 insX (22.6%) [52]. Exon 20 insertions are located at the C-terminal end or, more often, in the loop of the EGFR fragment known as the C-helix, which is a crucial element that regulates the activation status of *EGFR* [52]. An insertion in exon 20 creates of a wedge at the so-called “pivot point” of the C-helix, resulting in a rigid structure that prevents the C-helix from being repositioned to its external position (inactive state) [53]. Thus, regardless of ligand binding, *EGFR* becomes locked in an active conformation. It has also been demonstrated that the insertion’s location has a fundamental impact on EGFR TKI sensitivity, or the lack thereof, as it influences the drug’s kinetic properties and ATP binding [50].

Mutations in the *HER2* gene, which encodes one of the tyrosine kinase receptors of the EGFR family, are much less common among NSCLC patients (2–4%); over 90% are exon 20 insertions. They are more frequent among non-smoking patients and women; the median age of the patients is approximately 60 years. These mutations may also lead to ligand-independent tyrosine kinase activation [54,55].

First-generation EGFR TKI (erlotinib, gefitinib) are unfortunately not effective in patients with the presence of *EGFR* exon 20 insertions; the rate of response to treatment oscillates from 8% to 27% and the median PFS does not exceed 3 months [56,57]. An analysis of data originating from the clinical studies LUX-Lung 2, LUX-Lung 3 and LUX-Lung 6 demonstrated that the response rate among the 23 patients with the presence of exon 20 insertions treated with afatinib amounted to 8.7%, whereas the median PFS (mPFS) was similar to that of first-generation inhibitors and amounted to only 2.7 months [58]. An analysis of nearly 700 patients with uncommon *EGFR* gene mutations (other than deletion in exon 19 or substitution L858R in exon 21) treated with afatinib showed that the median DOR among patients in whom a response to afatinib was observed amounted to almost 12 months. The third-generation inhibitor osimertinib also shows activity with regard to some *EGFR* exon 20 insertions (mPFS: 6.2 months) [59].

#### 2.3.1. Poziotinib

Poziotinib is a new oral, irreversible EGFR TKI, which inhibits not only *EGFR*, but also *HER2* and *HER4*. In cells with *EGFR* exon 20 insertions, the drug-binding pocket is relatively small. Poziotinib is centered on a less rigid core than third-generation TKIs, whereas the molecule itself is smaller and more flexible than those of second- and third-generation inhibitors [54]. Its efficacy in NSCLC patients with molecular alterations within *HER2* or *EGFR* was assessed in the multicohort phase 2 clinical study ZENITH20 [60]. Cohort C2 included 90 pretreated patients with *HER2* exon 20 insertions; 67% of the patients had previously received at least two lines of treatment. Poziotinib was administered in the dose of 16 mg per day. The study’s primary endpoint was ORR, which amounted to 27.8%. The observed lower bound of 18.9% exceeded the pre-specified lower bound of 17%. The median DCR amounted to 70%.

The median DOR amounted to 5.1 months, and the median PFS was 5.5 months. The most frequent adverse effects were typical of this group of agents and included diarrhea (82% of the patients; grade ≥ 3: 26%), rash (68%; grade ≥ 3: 30%), stomatitis (66%; grade ≥ 3: 22%) and paronychia (38%; grade ≥ 3: one patient). Temporary interruptions of treatment due to adverse effects were necessary in 87% of patients, and the treatment was discontinued in 12% of the patients due to toxicity. In cohort C1, the drug’s efficacy and safety were assessed in pretreated patients with *EGFR* exon 20 insertions, but the study failed to meet its primary endpoint [61]. The primary endpoint was also not met in cohort C3, which included patients with *EGFR* exon 20 insertions who had not received previous treatment. Although the pre-specified lower bound of a RR > 20% (95% CI) was not met, the ORR was 27.8% among the 79 patients enrolled in this cohort. Detailed results of the ZENITH 20 study for cohorts C2 and C3 are presented in Table 3.

#### 2.3.2. Mobocertinib

Mobocertinib (TAK-788) is a low-molecular-weight irreversible EGFR TKI designed to selectively target *EGFR* or *HER2* exon 20 insertions rather than the whole epidermal growth factor receptors family. The efficacy and safety of mobocertinib in previously treated lung cancer patients was assessed in a phase 1/2 clinical study, EXCLAIM [62]. Its maximum tolerated dose, as assessed in the study’s extension cohort, was 160 mg. The second phase of the study included 28 patients with *EGFR* exon 20 insertions. The study’s primary endpoint was ORR, which amounted to 43%; DCR proved to be twice as high (86%). The median DOR amounted to 13.9 months (95% CI 5.0–NR), and the median PFS exceeded 7 months (mPFS 7.3 months). The drug’s toxicity profile was assessed in a group of 72 patients receiving 160 mg per day. It is consistent with the profile of the whole group of EGFR inhibitors, and the adverse effects are amenable to symptomatic treatment. The most frequent adverse effects involved the gastrointestinal tract and included diarrhea (82% of patients; grade 3 or higher according to CTCAE: 32%), nausea (39%; grade ≥ 3: 11%), vomiting (36%; grade ≥ 3: 7%) and appetite loss (39%). Acneiform rash, typical of this class of medications, occurred in 46% of patients, but its intensity did not exceed grade 2 [62] in any of the patients. The ongoing phase 3 clinical trial EXCLAIM-2 was designed to compare the efficacy and safety of mobocertinib with standard first-line platinum-based chemotherapy in previously untreated patients. To date, the median length of PFS in patients with *EGFR* exon 20 insertions receiving standard chemotherapy did not exceed 7 months; 1-year survival was achieved by 15–30% of the patients [63]. Results of this study will hopefully bring another treatment option in first line treatment in this poorly prognostic genetic alteration. The FDA granted breakthrough therapy for mobocertinib for the treatment of NSCLC patients with *EGFR* exon 20 insertions following platinum-based chemotherapy failure (27 April 2020).

#### 2.3.3. Amivantamab

Amivantamab is a fully-human antibody targeting EGFR and MET; its activity has been demonstrated in patients with *EGFR* exon 19 deletions, L858R substitutions, T790M and C797S mutations and *EGFR* exon 20 insertions, as well as in patients with mutations and amplifications in the *MET* gene. Its efficacy and safety in patients with *EGFR* exon 20 insertions was assessed in the phase 1 clinical trial CHRYSALIS [64]. The dose recommended for evaluation in phase 2 of the study was 1050 mg per day (1400 mg for patients ≥80 kg). In the course of the study, amivantamab was administered to 50 patients, 58% of whom had previously received platinum-based chemotherapy. The ORR amounted to 36% (41% for patients treated with platinum). The median DOR among the patients who responded to treatment lasted 10 months, and the mPFS was 8.3 months. The most frequent adverse events were typical of this group of agents and included rash (72% of the patients), infusion-related reactions (60%), paronychia (34%) and stomatitis (16%). Grade ≥ 3 adverse reactions (CTCAE) were reported in 36% of the patients. In March 2020, the FDA designated amivantamab as a breakthrough therapy for patients with *EGFR* exon 20 insertions in whom the disease progressed during or after platinum-based chemotherapy.

#### 2.3.4. Trastuzumab deruxtecan

Trastuzumab deruxtecan (T-DXd) is an antibody-drug conjugate consisting of a humanized anti-HER2 antibody (class: IgG1), a cleavable tetrapeptide-based linker, and a cytotoxic topoisomerase 1 inhibitor. In a multicohort phase 1 clinical study including patients with various solid tumors, the ORR among HER2-positive NSCLC patients was 72.7% (eight out of 11 patients), whereas the mPFS was 11.3 months (95% CI 8.1–14.3 months). The phase 2 trial DESTINY-Lung01 was designed to include 90 patients with non-squamous lung cancer with *HER2* mutations and 80 patients with HER2 overexpression [65]. The patients received trastuzumab deruxtecan dosed at 3.6 mg/kg of body weight every 3 weeks in the form of intravenous infusions. Among the 42 patients with *HER2* mutations, the ORR amounted to 61.9% (95% CI 45.6–76.4), the DCR was 90.5% (95% CI 77.4–95.3), the median DOR was not reached at the data cutoff, whereas the estimated mPFS was 14 months. The most frequent adverse events included nausea, hair loss, anemia, appetite loss, reduced neutrophil count, vomiting and diarrhea; only a small percentage were reported as grade ≥ 3 events. In May 2020, the FDA designated the drug as a breakthrough therapy for NSCLSC patients with *HER2* mutations after failed platinum-based treatment.

### 2.4. Ret Pathway Inhibitors

The *RET* gene is a proto-oncogene encoding a transmembrane tyrosine kinase receptor, which consists of an intracellular kinase, a large extracellular domain and a transmembrane domain. The *RET* gene participates in normal embryonic development. The binding of the receptor with a ligand results in autophosphorylation of the intracellular tyrosine kinase, activation of signaling pathway transmission, cellular proliferation, an increase in cellular invasiveness and the development of metastases. Molecular alterations in the *RET* gene result in abnormal expression of kinase proteins and constitutive, ligand-independent activation of signaling pathways, leading to carcinogenesis [66,67]. Alterations leading to the activation of oncogenesis most commonly consist in mutations (37% of all alterations), fusions (31%) and amplifications (25%) [68,69]. *RET* gene fusions occur in 1–2% of NSCLC patients [70]. *RET* gene fusions occur more frequently among younger individuals and non-smokers, regardless of the patient’s sex; they are found almost exclusively among patients diagnosed with adenocarcinoma (most often papillary) [71]. The presence of *RET* fusions is associated with a high risk of intracranial dissemination—CNS metastases are found at diagnosis in approximately 25% patients with stage IV disease, and the lifetime prevalence of brain metastases is estimated at 46% [72]. The effectiveness of multikinase inhibitors, such as cabozantinib or vandetanib, in patients with *RET* alterations is limited, as they inhibit non-RET kinases and are characterized by relatively high toxicity.

#### 2.4.1. Selpercatinib

Selpercatinib (LOXO-292) is a competitive, highly selective, low-molecular-weight inhibitor of RET tyrosine kinase, which exhibits activity both with regard to fusions and activating point mutations. It was also designed to successfully overcome the blood–brain barrier and achieve high concentrations in the CNS. Its efficacy and safety in patients with RET-positive NSCLC was confirmed in the clinical study LIBRETTO-001 [73]. This phase 1/2 study included patients with solid tumors harboring *RET* alterations. The study recruited 105 lung cancer patients who had previously received at least one line of platinum-based treatment; over half of the patients had received prior programmed death ligand (PD-L1) inhibitors (55%), and nearly half had received multikinase inhibitors (48%). The study also included 56 treatment-naive patients. The study’s primary endpoint was ORR. In patients who had received prior systemic treatment, the ORR was 64%; a complete response was reported in 2% of the patients, and a partial response in 62%. The response to treatment did not depend on the type of previous treatment (immunotherapy or TKI) or on the fusion partner of the *RET* gene. Median DOR was 17.5 months. After the observation period, with a median duration of approximately 12 months, 63% continued to respond to treatment, and the mPFS was 16.5 months according to an independent review committee. Among the patients who had received prior systemic treatment, 38 (36%) had CNS metastases at inclusion; 11 patients had measurable lesions according RECIST 1.1. During the course of the treatment, the rate of intracranial response amounted to 91%, which indicates that selpercatinib has very high intracranial activity. Among the previously untreated patients, the rate of treatment response amounted to 85% according to independent review and 90% according to investigator assessment. The most frequent adverse events classified as grade 3 or 4 events (CTCAE) included hypertension (14% of the patients), increased alanine transaminase activity (13%), increased aspartate aminotransferase activity (10%), hyponatremia (6%) and lymphopenia (6%). The results concerning adverse events and the rate of their occurrence in NSCLC patients were similar to those obtained among all 531 patients with solid tumors receiving selpercatinib as part of the LIBRETTO-001 study. In May 2020, the FDA approved selpercatinib for the treatment of metastatic RET-fusion positive NSCLC and metastatic RET-mutant medullary thyroid cancer. Currently underway is the open-label phase 3 trial LIBRETTO-431, comparing the efficacy of selpercatinib to that of standard chemotherapy (cisplatin/carboplatin + pemetrexed) in combination with pembrolizumab in patients with RET-positive NSCLC [74]. The detailed results of selpercatinib efficacy are presented in the Table 4.

#### 2.4.2. Pralsetinib

Pralsetinib (BLU-667) is another oral low-molecular-weight RET TKI with confirmed efficacy in RET-positive NSCLC which has been approved by the FDA. ARROW, an open-label multicohort phase 1/2 clinical trial, included 87 previously treated lung cancer patients and 27 patients who had received no prior treatment [75]. Among the patients receiving pralsetinib as a next line of treatment, the ORR was 57%; the response rate was higher among previously untreated patients (70%). After 6 months, the response to pralsetinib treatment persisted in 56% of previously untreated patients and in 80% of patients who had received prior treatment. The efficacy was independent of the fusion partner or the presence of CNS metastasis. Safety analysis, performed as part of the study, evaluated the data of 354 patients. The most frequent adverse events of pralsetinib included increased aspartate aminotransferase activity (31% of the patients), reduced hemoglobin concentration (22%), increased alanine aminotransferase activity (21%), constipation (21%), hypertension (20%), as well as lymphopenia and neutropenia. In September 2020, selpercatinib was approved by the FDA for the treatment of patients with RET-positive NSCLC. The detailed results of pralsetinib efficacy are presented in the Table 4.

## 3. Conclusions

The prognosis for patients with stage IV lung cancer continues to be poor. Improving the prognosis for patients with NSCLC (especially adenocarcinoma) hinges on introducing targeted therapies. The use of molecularly targeted therapies is based on molecular diagnostics, which requires the acquisition of an appropriate amount of good-quality tissue samples. It is now common to use EGFR TKIs in patients with activating mutations within the *EGFR* gene (10–15% of Caucasian NSCLC patients), ALK TKIs in patients with *ALK* gene rearrangements (5–7%), ROS1 TKIs in patients with *ROS1* gene rearrangements/fusions (1–2%) and dabrafenib in combination with trametinib in patients with *BRAF* gene mutations (2–4%). Recent years have witnessed the discovery of significant signaling pathways causing NSCLC development and progression, for which efficacious drugs have been designed. Alterations in *MET* (3–4% of NSCLC patients), *NTRK* (3%), *HER2* (2–4%) and *RET* (1–2%) genes, as well as *EGFR* exon 20 insertions (1–2%) are rare and mutually exclusive molecular aberrations. Nevertheless, when all common and rare molecular alterations in NSCLC are considered together, it turns out that 30–50% of patients harboring these alterations could be successfully treated with targeted therapies. Patients with molecularly targetable disease have a chance of avoiding chemotherapy in their first line treatment, and importantly, this treatment is well tolerated, giving them a chance to lead a normal family, social and professional life. Targeted therapies are also active in the CNS and may postpone brain radiotherapy, which is especially important for younger patients. Searching for molecular aberrations and implementing targeted therapy if appropriate should be a key diagnostic approach for NSCLC patients.

## Figures and Tables

**Table 1:** Detailed results of mesenchymal epithelial transition (MET) tyrosine kinase inhibitor (TKI) efficacy. Abbreviations: DCR, disease control rate; mDOR, median duration of response; ORR, overall response rate; mPFS, median progression-free survival.

**Capmatinib (INC280): Orally, 2 × 400 mg Per Day FDA Approval 6 May 2020**														
GEOMETRY NCT02414139 n = 364	ORR		Failure of 1–2 lines of therapy	Treatment-naive	mDOR			Failure of 1–2 lines of therapy		Treatment-naive	mPFS		Failure of 1–2 lines of therapy	Treatment-naive
		METex14mut	41% 95% CI 29–53%	68% 95% CI 48–84%			METex14mut	9.7 mths 95% CI 5.6–13 mths		12.6 mths 5.6 mths-NE		METex14mut	5.4 mths 95% CI 4.2–7.0 mths	12.4 mths 95% CI 8.2 mths-NE
		MET amplification	29% 95% CI 19–41%	40% 95% CI 16–68%			MET amplification					MET amplification	4.1 mths 95% CI 2.9–4.8 mths	4.2 mths 95% CI 1.4–6.9 mths
**Tepotinib: orally, 1 × 500 mg per day March 2020 approval in Japan**														
VISION NCT02864992 n = 152						ORR			mDOR			mPFS		
		Combined biopsy group				46% 95% CI 36–57%			11.1 mths 95% CI 7.7 mths—NE			8.5 mths 95% CI 6.7–11 mths		
		Liquid biopsy				48% 95% CI 36–65%			9.9 mths 95% CI 7.7 mths—NE			8.5 mths 95% CI 5.1–11 mths		
		Tissue biopsy				50% 95% CI 37–63%			15.7 mths 95% CI 9.7 mths—NE			11 mths 95% CI 5.7–17.1 mths		
**Savolitinib: orally, 1 × 600 mg per day (<50 kg: 400 mg) not yet approved**														
NCT02897479 n = 87		ORR				DCR			mDOR			mPFS		
		47.5% (95% CI 34.6–60.7%)				93.4% (95% CI 84.1–98.2%)			NR			6.8 mths (95% CI 4.2–13.8 mths)		

**Table 2:** Detailed results of NTRK TKI efficacy.

	STUDY	mDOR	mPFS	ORR
Entrectinib	ALKA-372-001, STARTRK-1, STARTRK-2	12.9 (7.9–NE),	11.2 (8.0–14.9)	59.3% (45.0–72.4) 70% (35–93) with NSCLC
Larotrectinib	NCT02122913, NCT02637687 NCT02576431	35.2 (22.8–NE)	25.8 (CI 9.9–NE)	79% (72–85) 75% (43–95) with NSCLC
Selitrectinib	NCT03215511	N/A	N/A	34% (10 out of 29)
Repotrectinib	NCT03093116	1.7+ to 3.6+ months with all 3 patients remaining in a response at the time of the data cutoff.		50% (12–88) 3 out of 6 patients with NTRK fusion
Taletrectinib	NCT02279433 NCT0267549 NCT04617054 (not yet recruiting)			66.7% (35.4–87.9) treatment naive ROS + NSCLC33.3% patients with crizotinib-refractory ROS1 + NSCLC. One patient with NTRK1 differentiated thyroid cancer achieving a confirmed partial response of 27 months at data cutoff.

**Table 3:** Detailed results of the ZENITH 20 study for cohorts C2 and C3.

Poziotinib: Orally, 1 × 16 mg Per Day			
ZENITH20 NCT03318939 n = 90 (cohort 2) n = 79 (cohort 3)		Cohort 2 HER2ex20ins, previously treated	Cohort 3 EGFRex20ins, treatment-naive
	ORR	27.8% 95% CI 18.9–38.2% pre-specified lower bound of 95% CI > 17% met	27.8% 95% CI 18.4–39.1% pre-specified lower bound of 95% CI > 20% not met
	mDOR mFU 8.3 mths	5.1 mths 95% CI 1–12.3 mths	
	DCR	63%	86.1%
	mPFS	5.5 mths 95% CI 0–13.1 mths	7.2 mths
	dose interruptions due to toxicity	87%	94%
	dose discontinuation due to toxicity	12%	8%

**Table 4:** The detailed results on selpercatinib and pralsetinib efficacy.

**Selpercatinib (LOXO-292): Orally, 2 × 120 mg Per Day (<50 kg), 2 × 160 mg Per Day (≥50 kg) FDA Approved 8 May 2020**					
LIBRETTO-001 NCT04194944 n= 161		PREVIOUSLY TREATED		TREATMENT-NAIVE	
		Independent committee review	Investigator assessment	Independent committee review	Investigator assessment
	ORR	64% 95% CI 54–73%	70% 95% CI 60–78%	85% 95% CI 70–94%	90% 95% CI 76–97%
	mDOR	17.5 mths 95% CI 12–NE	20.3 mths 95% CI 15.6–24 mths	NR	
	1yPFS	66%	68%		
	mPFS	16.5 mths 95% CI 13.7–NE	18.4 mths 95% CI 16.4–24.8 mths		
	icORR	91% 95% CI 89–100%			
	m icDOR	10.1 mths 95% CI 6.7–NE			
**Pralsetinib (BLU-667): orally, 1 × 400 mg per day (on an empty stomach) FDA approved 9 April 2020**					
ARROWN CT03037385 n = 114		PREVIOUSLY TREATED		TREATMENT-NAIVE	
	ORR	57% (95% CI 46–68%)		70% (95% CI 50–86%)	

## Data Availability

The datasets used and/or analyzed during the current study are available from the corresponding author on reasonable request.
